# Supplementary material for: Combined Effect of Caspase-Dependent and Caspase-Independent Apoptosis in the Anticancer Activity of Gold Complexes with Phosphine and Benzimidazole Derivatives
Source: Pharmaceuticals (Basel). 2020 Dec 24;14(1):10. doi: 10.3390/ph14010010 (PMC7824672; doi:10.3390/ph14010010)

Supplementary Materials for

Combined effect of caspase-dependent and caspase-independent apoptosis in the anticancer activity of gold complexes with phosphine and benzimidazole derivatives

Lara Rouco, Ángeles Sánchez-González*, Rebeca Alvariño*, Amparo Alfonso, Ezequiel M. Vázquez-López, Emilia García-Martínez, and Marcelino Maneiro*

Table of contents

Figure S1. ESI MS spectrum for **1**

Figure S2. ESI MS spectrum for **2**

Figure S3. UV-Vis spectrum for **1**

Figure S4. UV-Vis spectrum for **2**

Figure S5. IR spectrum for **1** at 4000-500 cm^{-1} (upper figure), and at 500-100 cm^{-1} (lower figure)

Figure S6. IR spectrum for **2** at 4000-500 cm^{-1} (upper figure), and at 500-100 cm^{-1} (lower figure)

Figure S7. ^1H NMR spectrum for **1** (registered in $CDCl_3$). Inset: details zoom to 7-9 ppm

Figure S8. ^1H NMR spectrum for **2** (registered in $CDCl_3$)

Figure S9. ^1H NMR spectrum for Hpben. Inset: details zoom to 7-9 ppm

Figure S10. ^{13}C NMR spectrum for Hpben

Figure S11. Heteronuclear Multiple Quantum Coherence (HMQC) correlation for Hpben

Figure S12. Heteronuclear Multiple Bond Correlation (HMBC) correlation for Hpben

Figure S13. ^{13}C NMR spectrum for **1** (registered in $CDCl_3$)

Figure S14. ^{31}P NMR spectrum for **1** (registered in $CDCl_3$)

Figure S15. ^{13}C NMR spectrum for **2** (registered in $CDCl_3$)

Figure S16. ^{31}P NMR spectrum for **2** (registered in $CDCl_3$)

Figure S17. MERCURY view for **1**

Figure S18. MERCURY view for **2**

Figure S19. Cytotoxicity curves for **1** and **2**

Checkcif files for the crystal structures of **1** and **2**

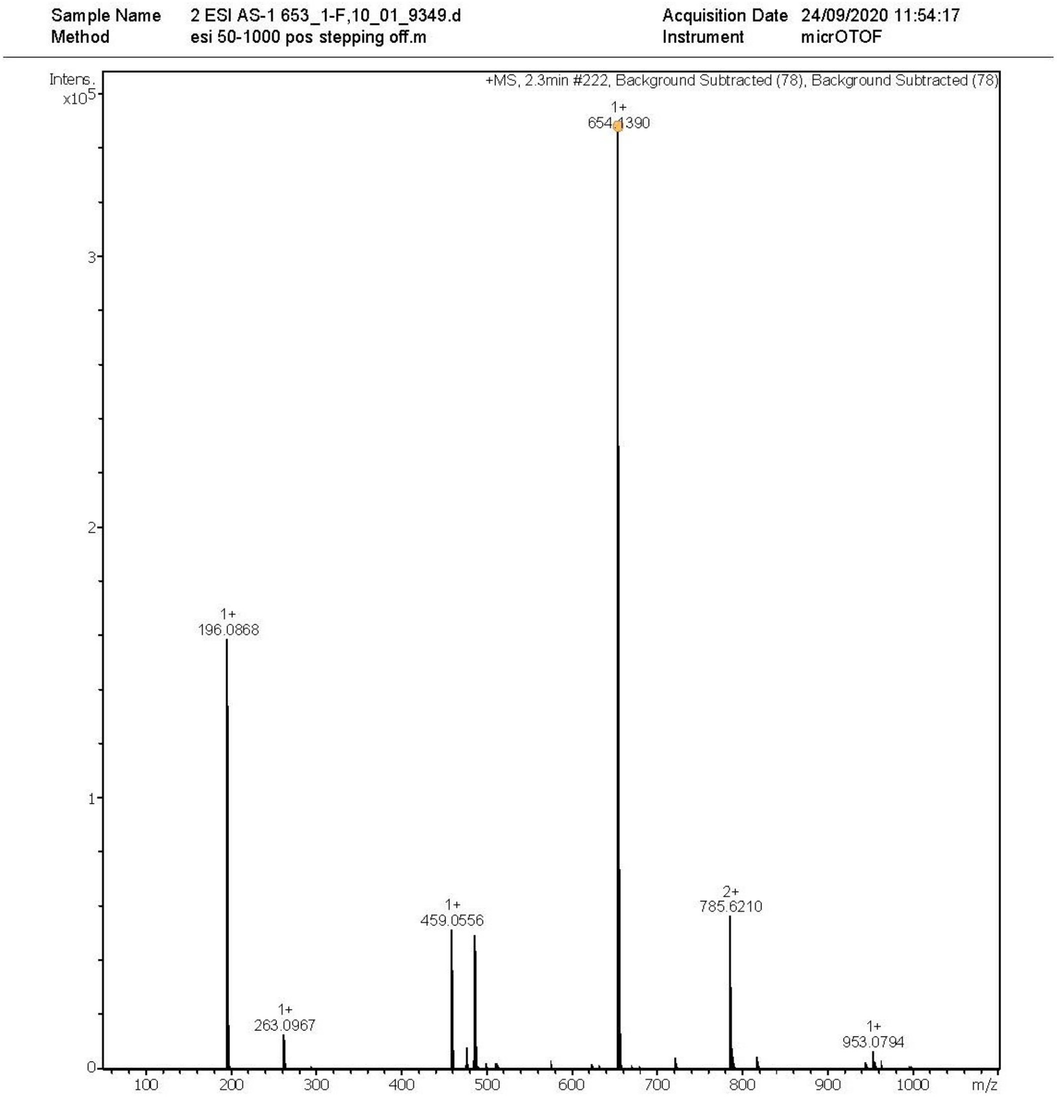

Figure S1. ESI MS spectrum for **1**

Analysis Info

Sample Name	3 ESI AS-2 509_1-G,11_01_9350.d	Acquisition Date	24/09/2020 12:03:31
Method	esi 50-1000 pos stepping off.m	Instrument	micrOTOF

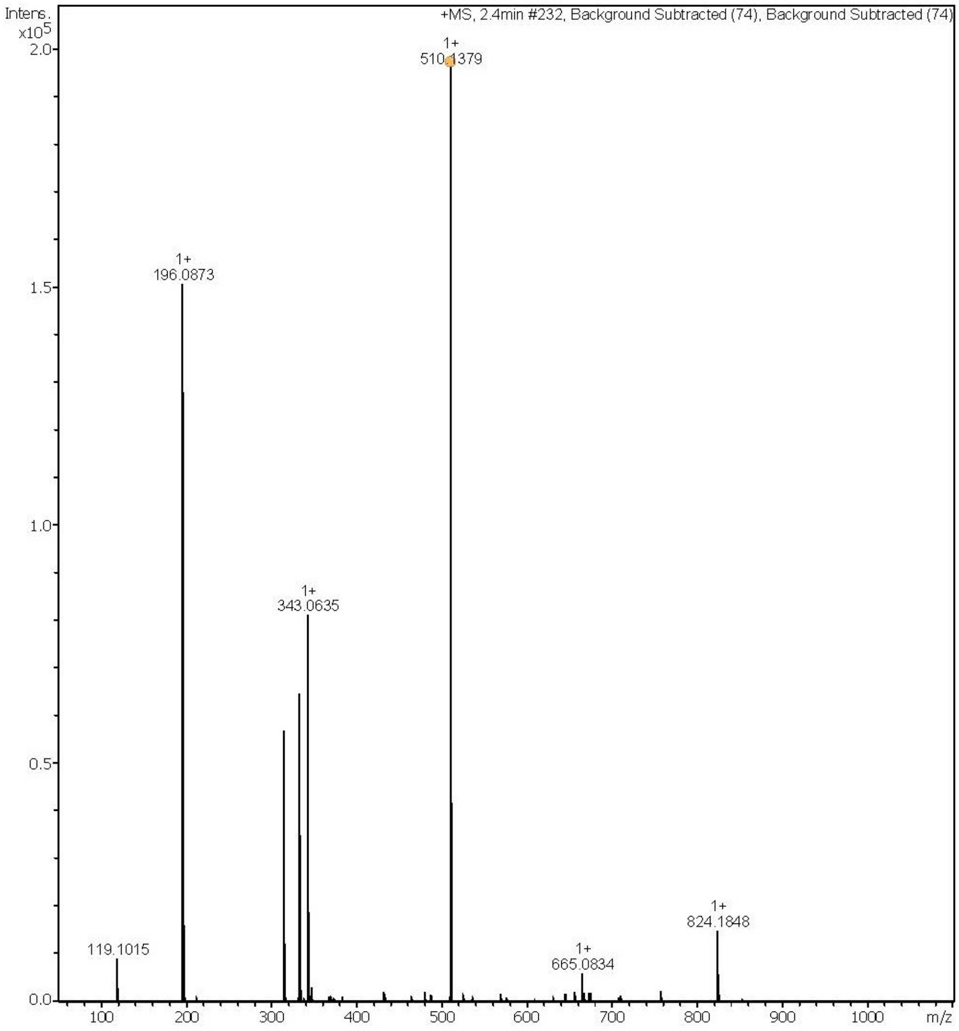

Figure S2. ESI MS spectrum for **2**

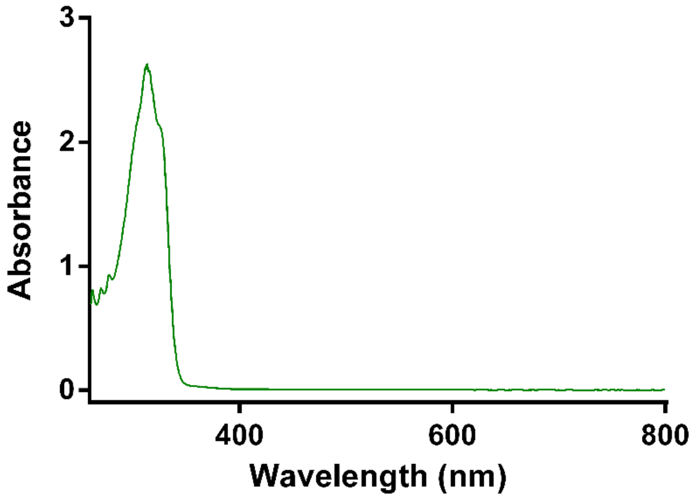

Figure S3. UV-Vis spectrum for **1**

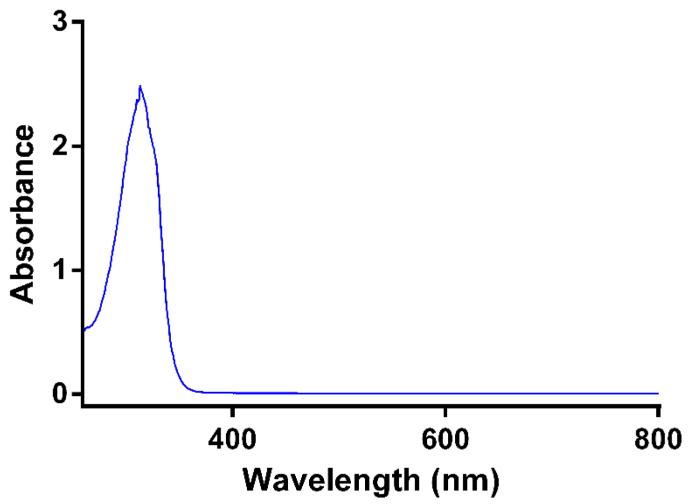

Figure S4. UV-Vis spectrum for **2**

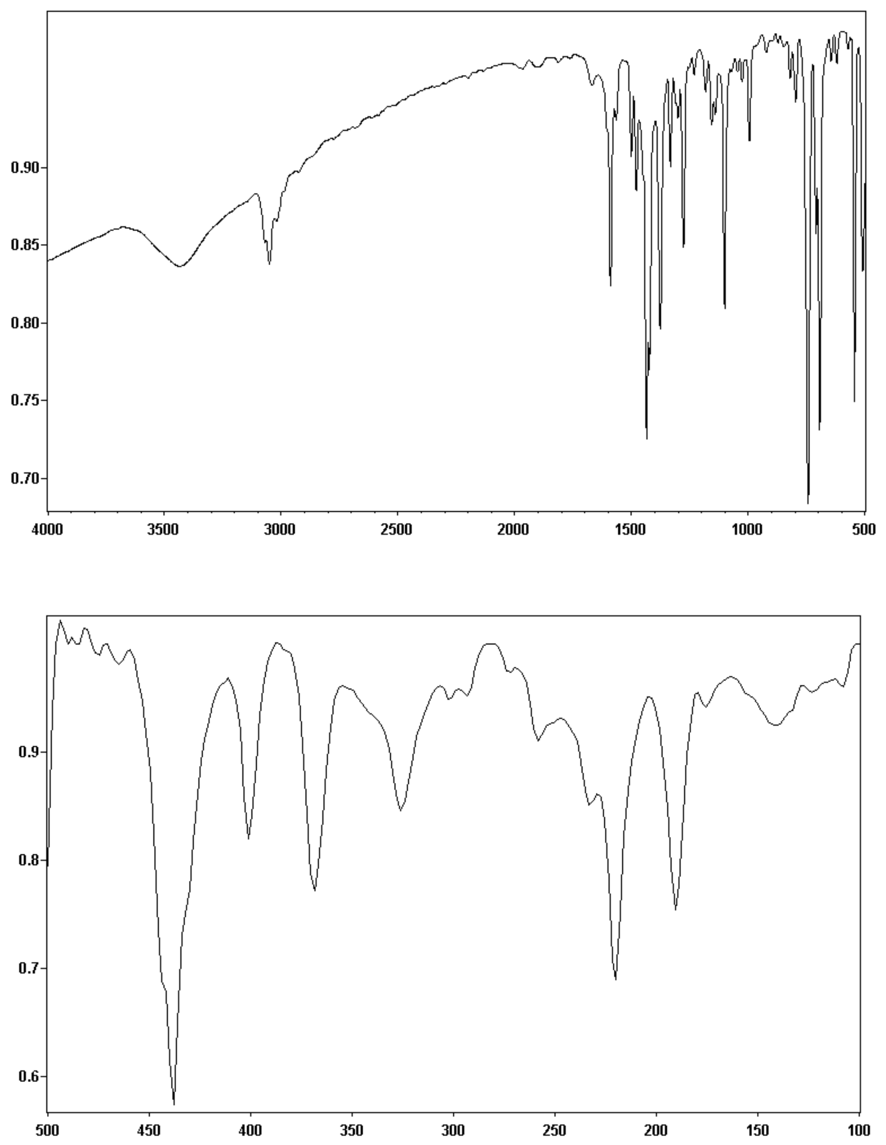

Figure S5. IR spectrum for **1** at 4000-500 cm^{-1} (upper figure), and at 500-100 cm^{-1} (lower figure)

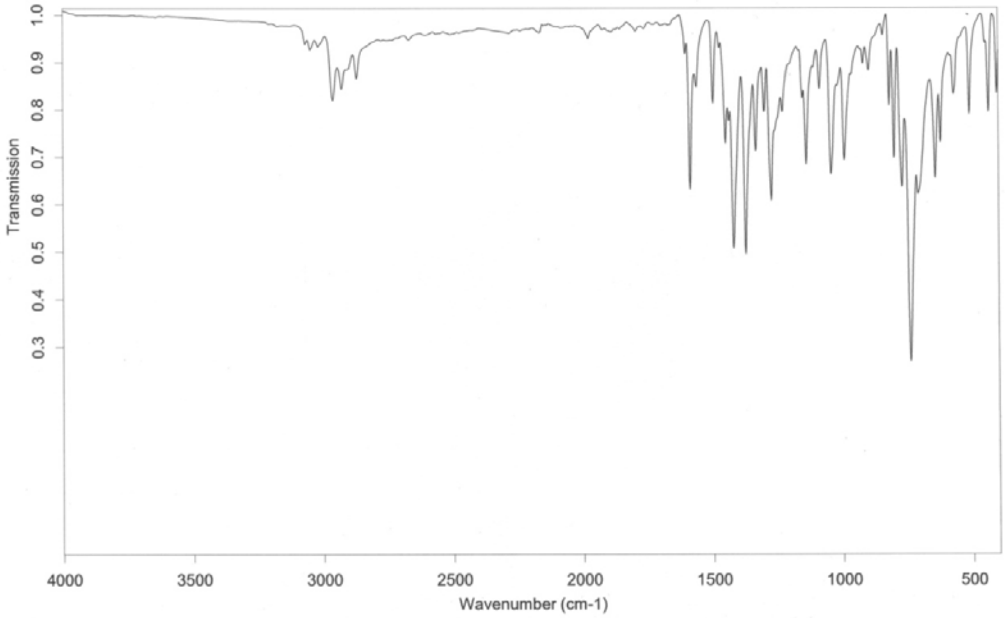

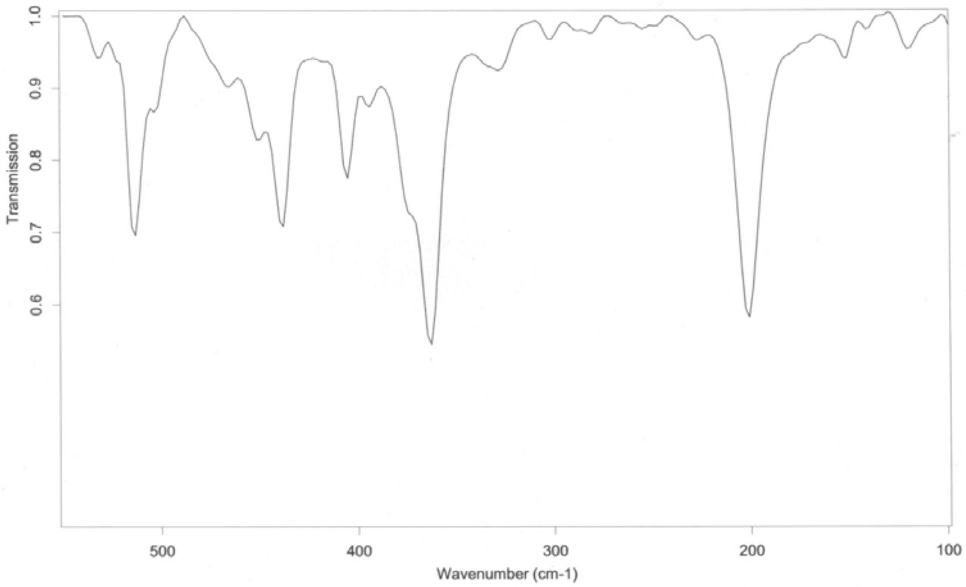

Figure S6. IR spectrum for **2** at 4000-500 cm^{-1} (upper figure), and at 500-100 cm^{-1} (lower figure)

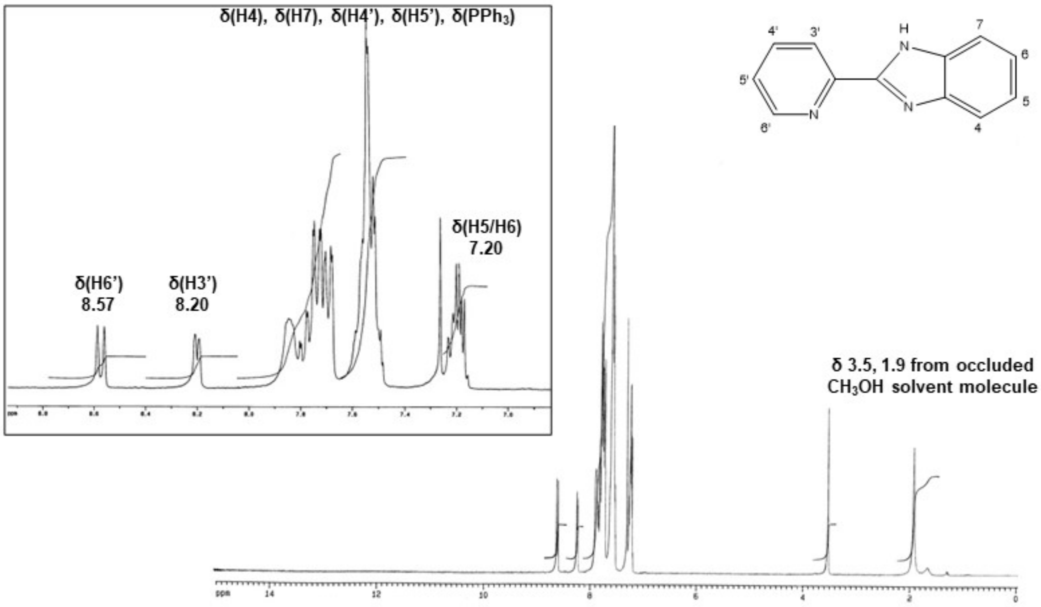

Figure S7. ^{1}H NMR spectrum for **1** (registered in $CDCl_3$). Inset: details zoom to 7-9 ppm

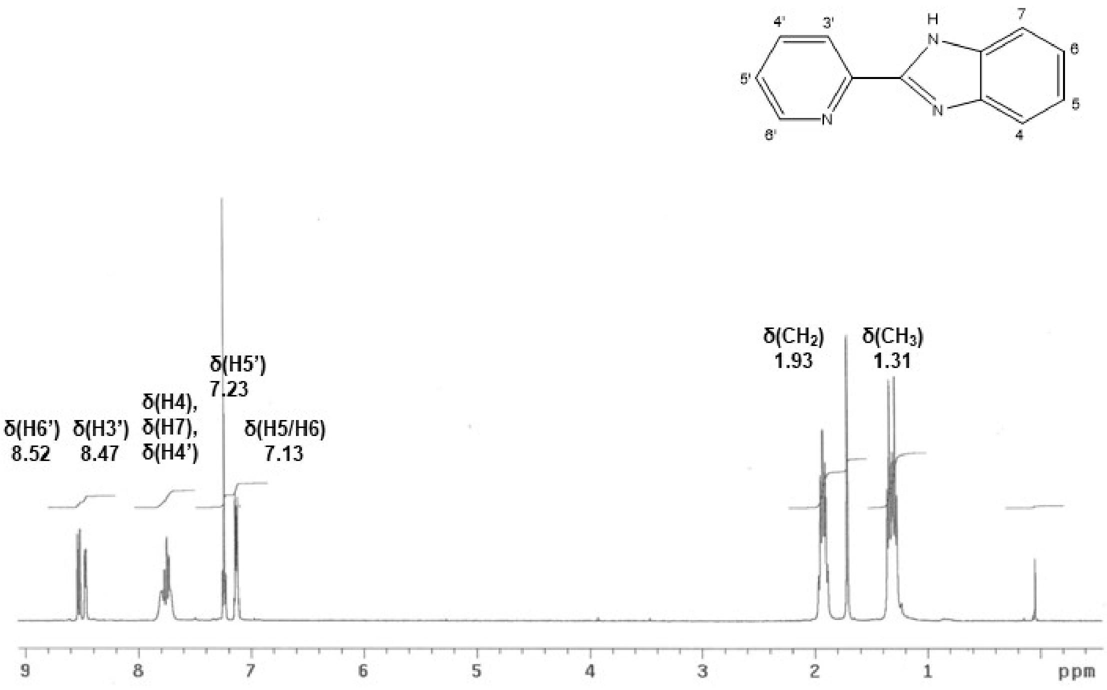

Figure S8. ^{1}H NMR spectrum for **2** (registered in $CDCl_3$), δ(CH_2) and δ(CH_3) from PEt_3 ligand.

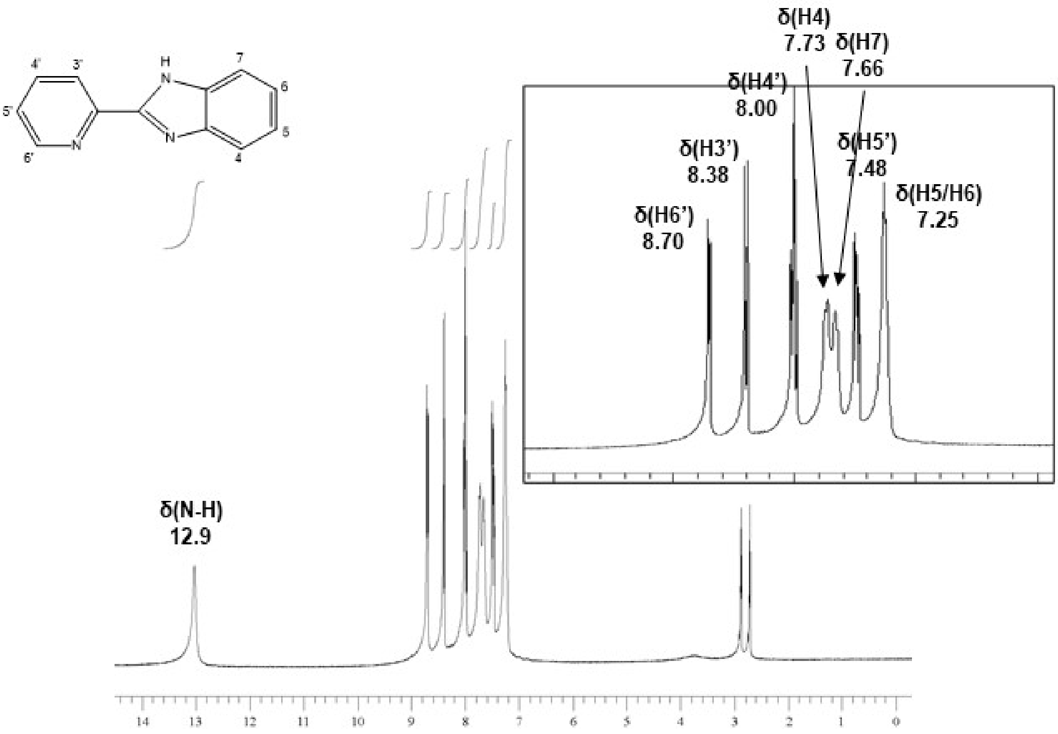

Figure S9. ^{1}H NMR spectrum for *Hpben*. Inset: details zoom to 7-9 ppm

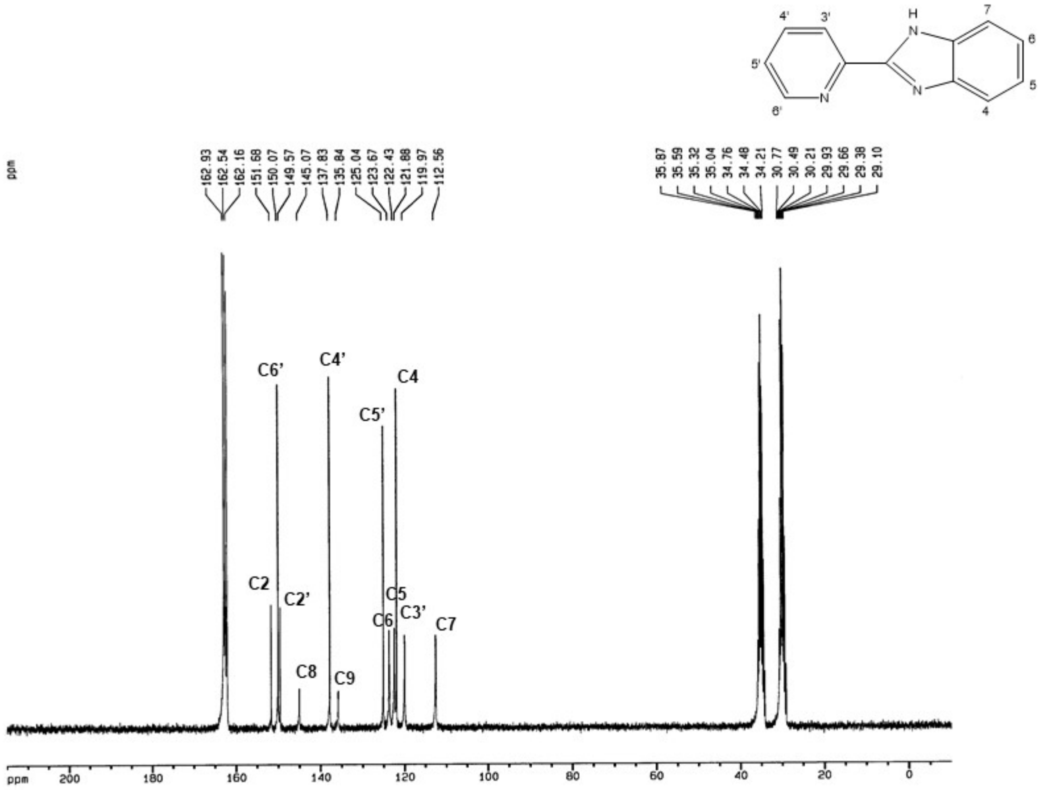

Figure S10. ^{13}C NMR spectrum for Hpben (in DMF-d7; 29-35 and 162 ppm signals are from DMF-d7).

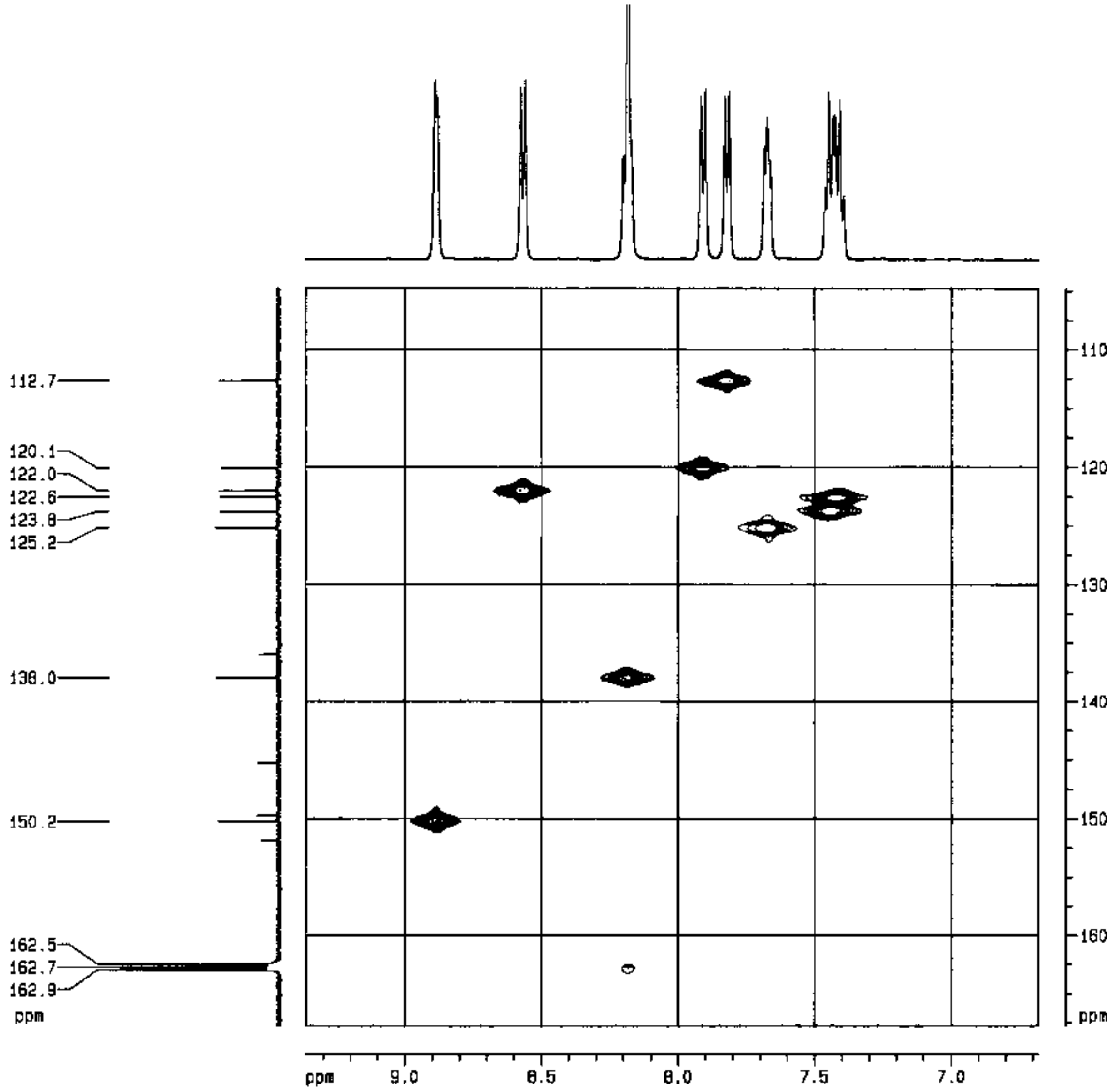

Figure S11. Heteronuclear Multiple Quantum Coherence (HMQC) correlation for Hpben

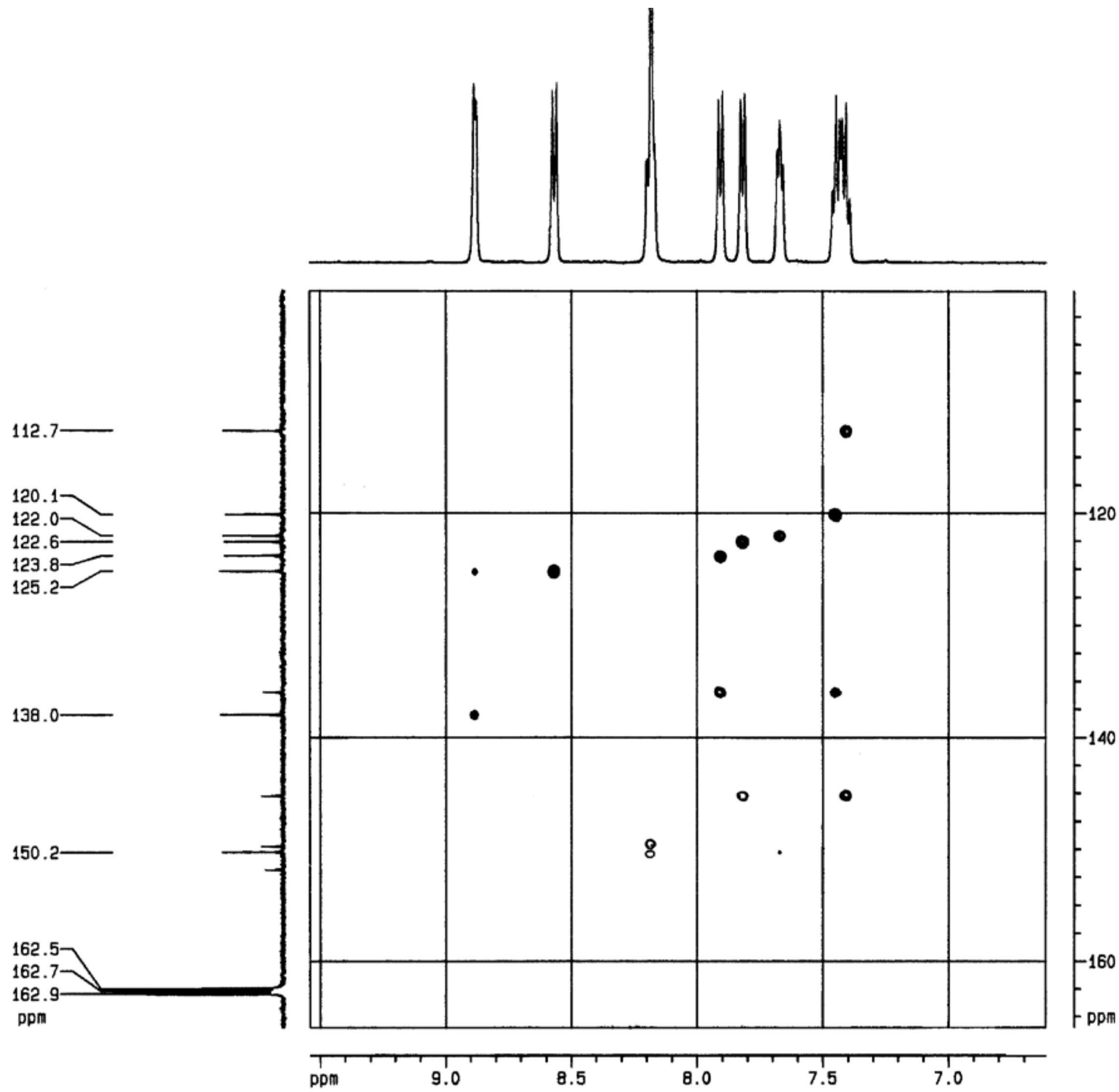

Figure S12. Heteronuclear Multiple Bond Correlation (HMBC) correlation for Hpben

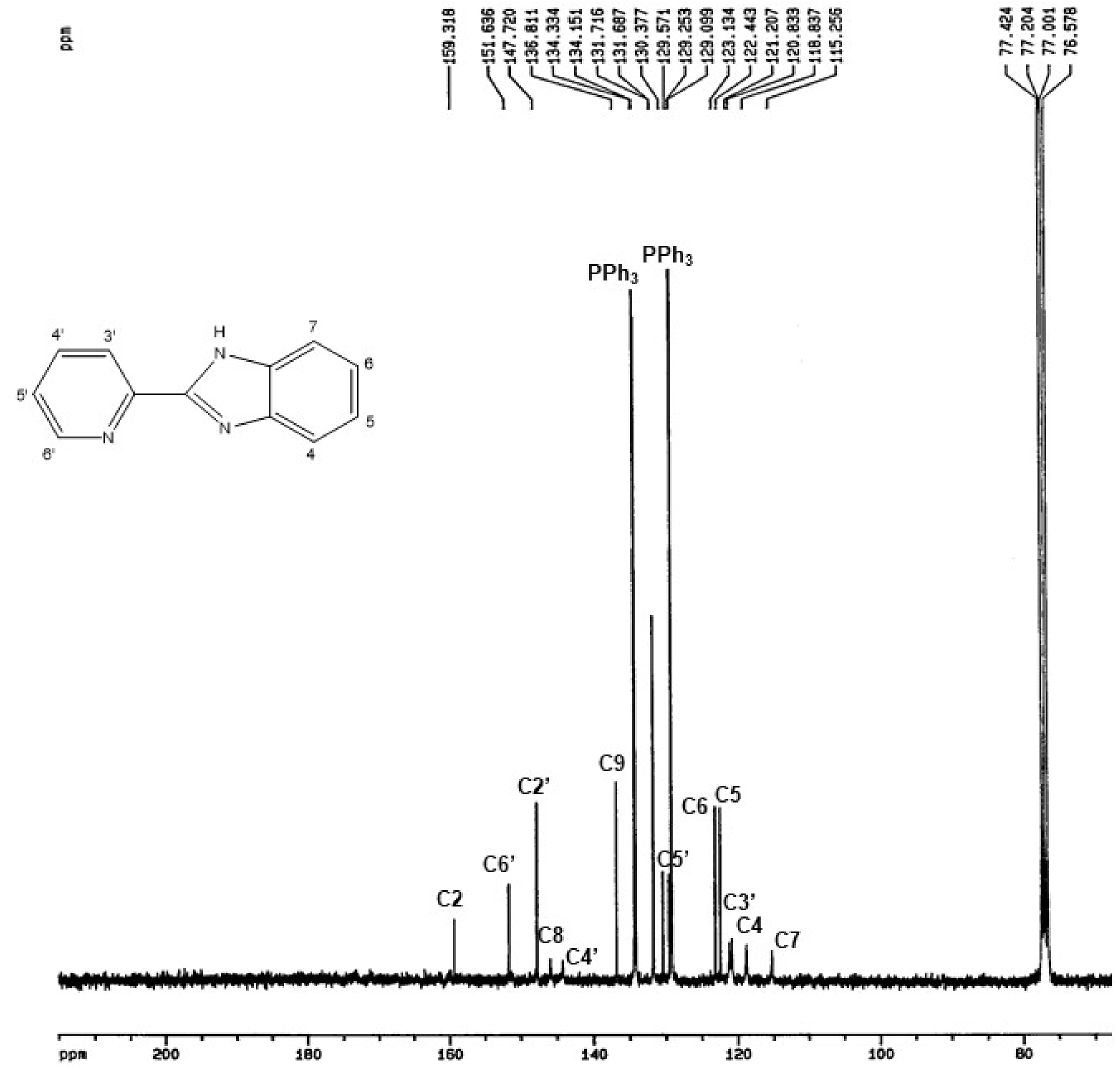

Figure S13. ^{13}C NMR spectrum for **1** (registered in $CDCl_3$)

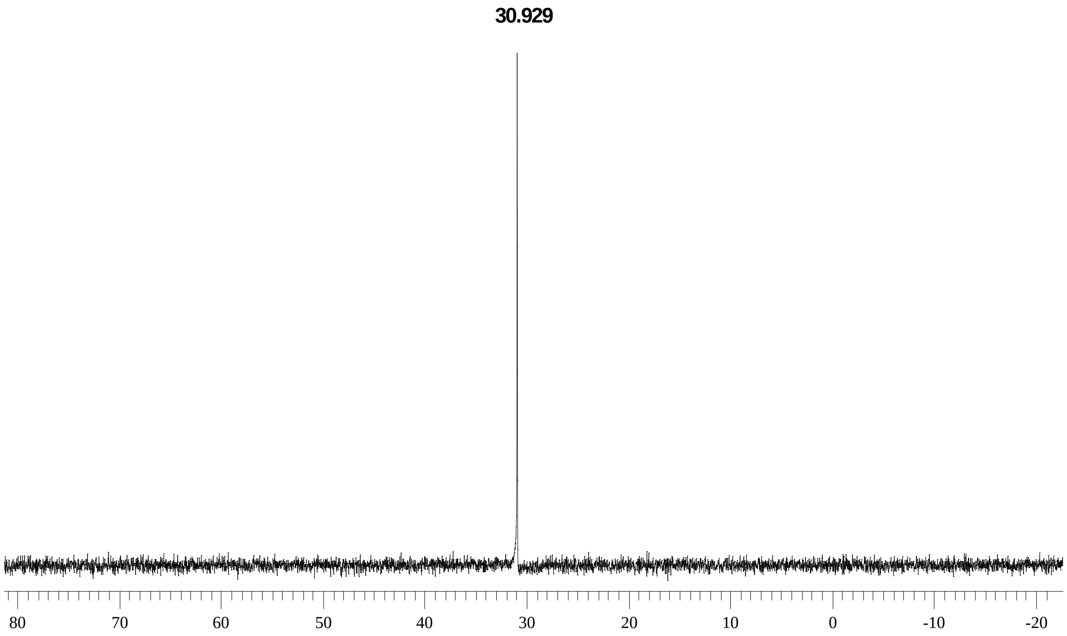

Figure S14. ^{31}P NMR spectrum for **1** (registered in $CDCl_3$)

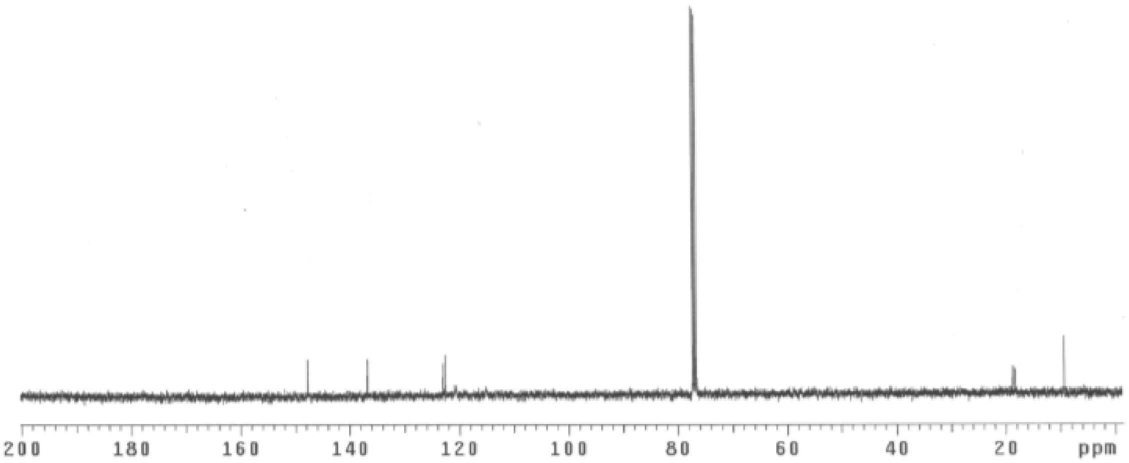

Figure S15. ^{13}C NMR spectrum for **2** (registered in $CDCl_3$)

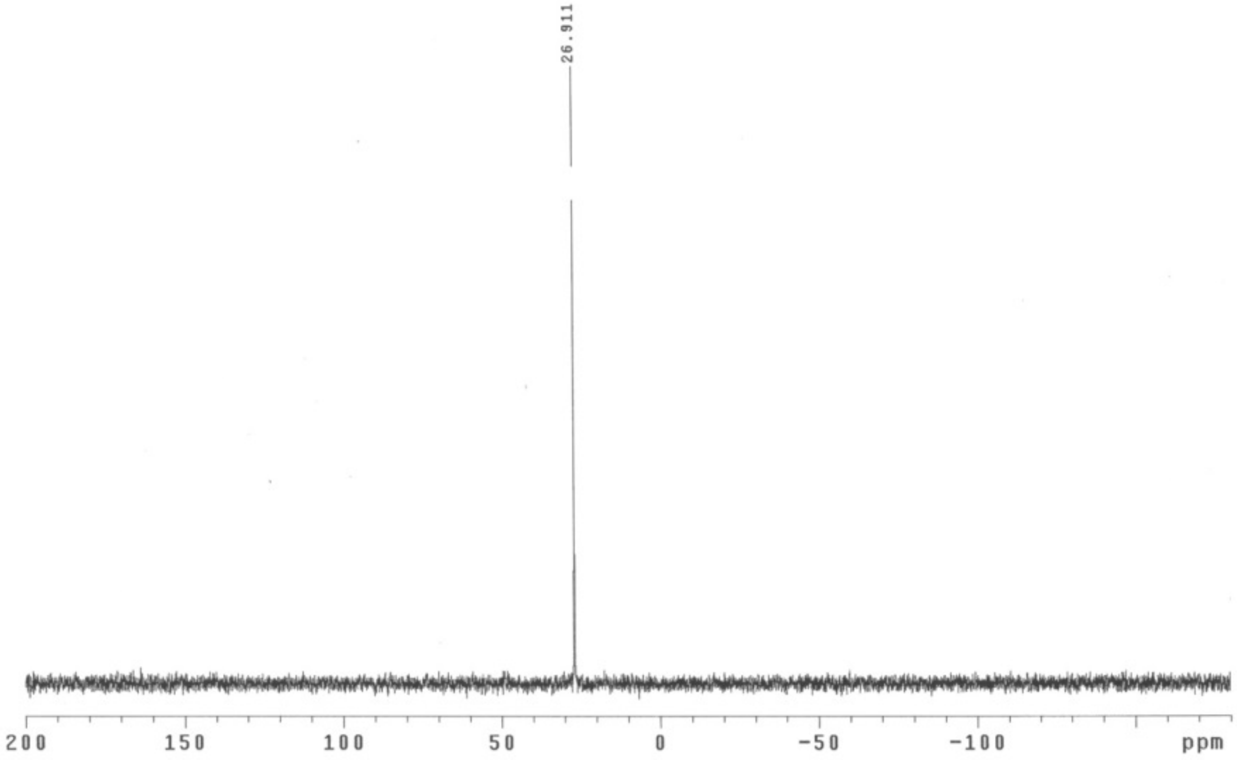

Figure S16. ^{31}P NMR spectrum for **2** (registered in $CDCl_3$)

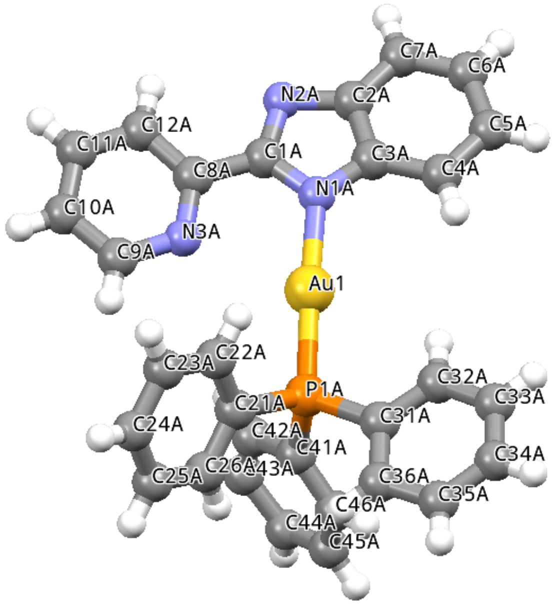

Figure S17. MERCURY view for **1**

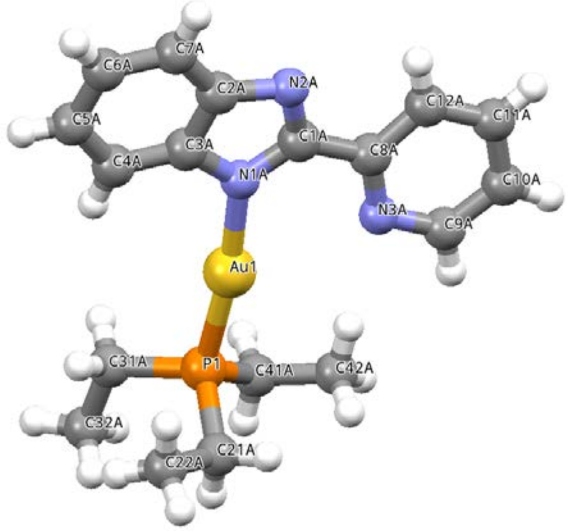

Figure S18. MERCURY view for **2**

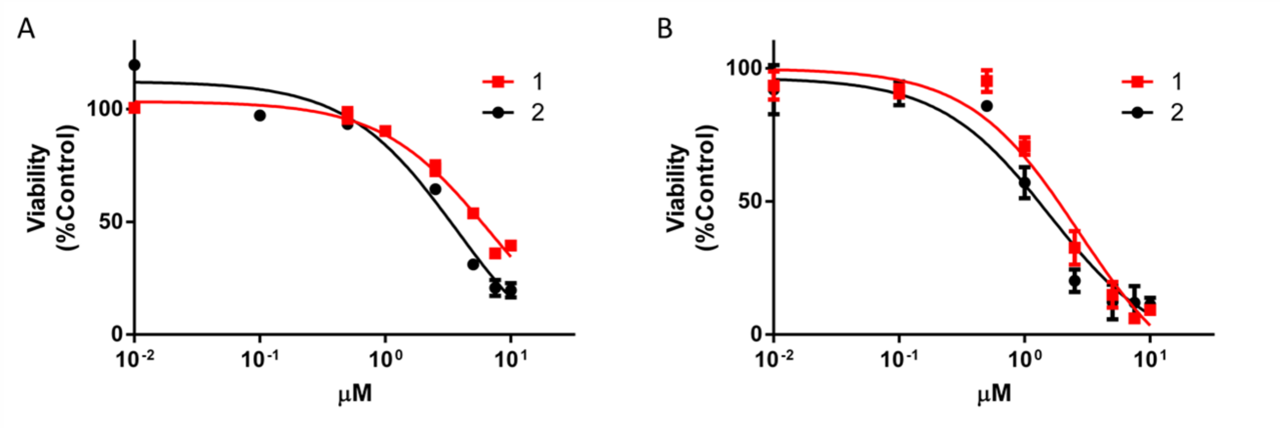

Figure S19. Cytotoxicity curves for **1** and **2**. A) incubation time 6h; B) incubation time 24 h.

checkCIF/PLATON report

Structure factors have been supplied for datablock(s) r86ms_jc_sq

THIS REPORT IS FOR GUIDANCE ONLY. IF USED AS PART OF A REVIEW PROCEDURE FOR PUBLICATION, IT SHOULD NOT REPLACE THE EXPERTISE OF AN EXPERIENCED CRYSTALLOGRAPHIC REFEREE.

No syntax errors found. CIF dictionary Interpreting this report

Datablock: r86ms_jc_sq

Bond precision: C-C = 0.0133 A Wavelength=0.71073

Cell:	a=9.4636(5)	b=18.3327(10)	c=32.0984(17)
	alpha=90	beta=98.278(1)	gamma=90
Temperature:	293 K		

	Calculated	Reported
Volume	5510.8(5)	5510.8(5)
Space group	P 21/c	P 21/c
Hall group	-P 2ybc	-P 2ybc
Moiety formula	C30 H23 Au N3 P [+ solvent]	?
Sum formula	C30 H23 Au N3 P [+ solvent]	C60 H46 Au2 N6 P2
Mr	653.45	1306.90
Dx,g cm-3	1.575	1.575
Z	8	4
Mu (mm-1)	5.419	5.419
F000	2544.0	2544.0
F000’	2529.99	
h,k,lmax	12,24,42	12,24,42
Nref	13324	12290
Tmin,Tmax	0.392,0.614	0.610,1.000
Tmin’	0.144	

Correction method= # Reported T Limits: Tmin=0.610 Tmax=1.000
AbsCorr = MULTI-SCAN

Data completeness= 0.922 Theta(max)= 28.005

R(reflections)= 0.0447(5740) wR2(reflections)= 0.0984(12290)

S = 0.807 Npar= 631

```
The following ALERTS were generated. Each ALERT has the format
       test-name_ALERT_alert-type_alert-level.
Click on the hyperlinks for more details of the test.
```

Alert level C

```
ABSTY02_ALERT_1_C  An _exptl_absorpt_correction_type has been given without
            a literature citation. This should be contained in the
            _exptl_absorpt_process_details field.
            Absorption correction given as multi-scan
PLAT026_ALERT_3_C Ratio Observed / Unique Reflections (too) Low ..        47% Check
PLAT029_ALERT_3_C _diffrn_measured_fraction_theta_full value Low .      0.960 Why?
PLAT234_ALERT_4_C Large Hirshfeld Difference C6A      --C7A      .       0.18 Ang.
PLAT234_ALERT_4_C Large Hirshfeld Difference C43A     --C44A     .       0.19 Ang.
PLAT234_ALERT_4_C Large Hirshfeld Difference C44A     --C45A     .       0.16 Ang.
PLAT234_ALERT_4_C Large Hirshfeld Difference C45A     --C46A     .       0.16 Ang.
PLAT234_ALERT_4_C Large Hirshfeld Difference N2B      --C1B      .       0.16 Ang.
PLAT234_ALERT_4_C Large Hirshfeld Difference C4B      --C5B      .       0.16 Ang.
PLAT234_ALERT_4_C Large Hirshfeld Difference C5B      --C6B      .       0.16 Ang.
PLAT234_ALERT_4_C Large Hirshfeld Difference C33B     --C34B     .       0.23 Ang.
PLAT234_ALERT_4_C Large Hirshfeld Difference C34B     --C35B     .       0.23 Ang.
PLAT234_ALERT_4_C Large Hirshfeld Difference C42B     --C43B     .       0.16 Ang.
PLAT234_ALERT_4_C Large Hirshfeld Difference C44B     --C45B     .       0.18 Ang.
PLAT241_ALERT_2_C High   'MainMol' Ueq as Compared to Neighbors of       C43A Check
PLAT241_ALERT_2_C High   'MainMol' Ueq as Compared to Neighbors of       C11B Check
PLAT342_ALERT_3_C Low Bond Precision on  C-C Bonds ...............    0.01334 Ang.
PLAT906_ALERT_3_C Large K Value in the Analysis of Variance ......     22.263 Check
PLAT906_ALERT_3_C Large K Value in the Analysis of Variance ......      5.631 Check
PLAT906_ALERT_3_C Large K Value in the Analysis of Variance ......      2.659 Check
PLAT911_ALERT_3_C Missing FCF Refl Between Thmin & STh/L=    0.600        399 Report
PLAT977_ALERT_2_C Check Negative Difference Density on H45B             -0.31 eA-3
```

Alert level G

```
PLAT045_ALERT_1_G Calculated and Reported Z Differ by a Factor ...       2.00 Check
PLAT199_ALERT_1_G Reported _cell_measurement_temperature ..... (K)        293 Check
PLAT200_ALERT_1_G Reported   _diffrn_ambient_temperature ..... (K)        293 Check
PLAT605_ALERT_4_G Largest Solvent Accessible VOID in the Structure        323 A**3
PLAT790_ALERT_4_G Centre of Gravity not Within Unit Cell: Resd.  #          2 Note
              C30 H23 Au N3 P
PLAT869_ALERT_4_G ALERTS Related to the Use of SQUEEZE Suppressed           ! Info
PLAT883_ALERT_1_G No Info/Value for _atom_sites_solution_primary .     Please Do !
PLAT910_ALERT_3_G Missing # of FCF Reflection(s) Below Theta(Min).          2 Note
PLAT912_ALERT_4_G Missing # of FCF Reflections Above STh/L=  0.600        634 Note
PLAT933_ALERT_2_G Number of OMIT Records in Embedded .res File ...          6 Note
PLAT941_ALERT_3_G Average HKL Measurement Multiplicity ...........        2.5 Low
PLAT961_ALERT_5_G Dataset Contains no Negative Intensities .......     Please Check
PLAT978_ALERT_2_G Number C-C Bonds with Positive Residual Density.          0 Info
```

```
   0 ALERT level A = Most likely a serious problem - resolve or explain
   0 ALERT level B = A potentially serious problem, consider carefully
  22 ALERT level C = Check. Ensure it is not caused by an omission or oversight
  13 ALERT level G = General information/check it is not something unexpected

   5 ALERT type 1 CIF construction/syntax error, inconsistent or missing data
   5 ALERT type 2 Indicator that the structure model may be wrong or deficient
   9 ALERT type 3 Indicator that the structure quality may be low
  15 ALERT type 4 Improvement, methodology, query or suggestion
   1 ALERT type 5 Informative message, check
```

It is advisable to attempt to resolve as many as possible of the alerts in all categories. Often the minor alerts point to easily fixed oversights, errors and omissions in your CIF or refinement strategy, so attention to these fine details can be worthwhile. In order to resolve some of the more serious problems it may be necessary to carry out additional measurements or structure refinements. However, the purpose of your study may justify the reported deviations and the more serious of these should normally be commented upon in the discussion or experimental section of a paper or in the "special_details" fields of the CIF. checkCIF was carefully designed to identify outliers and unusual parameters, but every test has its limitations and alerts that are not important in a particular case may appear. Conversely, the absence of alerts does not guarantee there are no aspects of the results needing attention. It is up to the individual to critically assess their own results and, if necessary, seek expert advice.

Publication of your CIF in IUCr journals

A basic structural check has been run on your CIF. These basic checks will be run on all CIFs submitted for publication in IUCr journals (*Acta Crystallographica*, *Journal of Applied Crystallography*, *Journal of Synchrotron Radiation*); however, if you intend to submit to *Acta Crystallographica Section C* or *E* or *IUCrData*, you should make sure that full publication checks are run on the final version of your CIF prior to submission.

Publication of your CIF in other journals

Please refer to the *Notes for Authors* of the relevant journal for any special instructions relating to CIF submission.

PLATON version of 18/09/2020; check.def file version of 20/08/2020

Datablock r86ms_jc_sq - ellipsoid plot

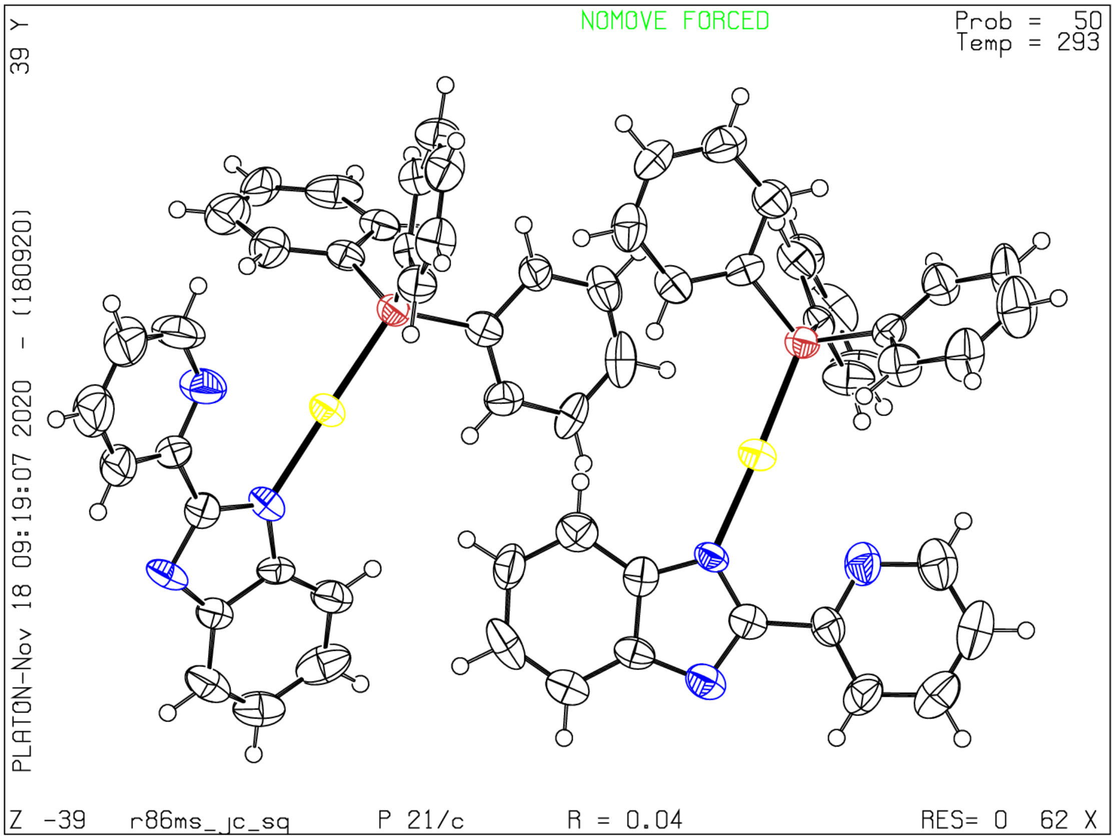

checkCIF/PLATON report

Structure factors have been supplied for datablock(s) ev161042_2_0m

THIS REPORT IS FOR GUIDANCE ONLY. IF USED AS PART OF A REVIEW PROCEDURE FOR PUBLICATION, IT SHOULD NOT REPLACE THE EXPERTISE OF AN EXPERIENCED CRYSTALLOGRAPHIC REFEREE.

No syntax errors found. CIF dictionary Interpreting this report

Datablock: ev161042_2_0m

```
Bond precision:  C-C = 0.0106 A          Wavelength=0.71073

Cell:           a=14.6206(10)    b=13.0799(9)       c=20.0179(14)
                alpha=90         beta=102.335(2)    gamma=90
Temperature:    100 K

                Calculated                 Reported
Volume          3739.8(4)                  3739.8(4)
Space group     P 21/n                     P 21/n
Hall group      -P 2yn                     -P 2yn
Moiety formula  C18 H23 Au N3 P            ?
Sum formula     C18 H23 Au N3 P            C18 H23 Au N3 P
Mr              509.33                     509.33
Dx,g cm-3       1.809                      1.809
Z               8                          8
Mu (mm-1)       7.956                      7.956
F000            1968.0                     1968.0
F000'           1954.04
h,k,lmax        19,17,26                   19,17,26
Nref            9413                       9342
Tmin,Tmax       0.097,0.135                0.092,0.234
Tmin'           0.073

Correction method= # Reported T Limits: Tmin=0.092 Tmax=0.234
AbsCorr = MULTI-SCAN

Data completeness= 0.992          Theta(max)= 28.423

R(reflections)= 0.0461( 7620)     wR2(reflections)= 0.0990( 9342)

S = 1.139                   Npar= 444
```

```
The following ALERTS were generated. Each ALERT has the format
       test-name_ALERT_alert-type_alert-level.
Click on the hyperlinks for more details of the test.
```

Alert level A

```
PLAT972_ALERT_2_A Check Calcd Resid. Dens.  0.72A   From Au2            -4.20 eA-3
```

Author Response: Although we were unable to determine the presence of several domains we did observe a strong dissagrement in a number of reflections with h<-11. We think that that and the unefficient absorption correction (rather usual in gold compound data) are responsible of the residual densities.

Alert level B

```
PLAT234_ALERT_4_B Large Hirshfeld Difference P1B      --C41B     .        0.26 Ang.
```

Author Response: This is consequence of the disorder in the PEt3 ligand.

```
PLAT234_ALERT_4_B Large Hirshfeld Difference C22B     --C21B     .        0.26 Ang.
```

Author Response: This is consequence of the disorder in the PEt3 ligand.

```
PLAT971_ALERT_2_B Check Calcd Resid. Dens.  0.70A   From Au2             3.45 eA-3
```

Author Response: Although we were unable to determine the presence of several domains we did observe a strong dissagrement in a number of reflections with h<-11. We think that that and the unefficient absorption correction (rather usual in gold compound data) are responsible of the residual densities.

```
PLAT971_ALERT_2_B Check Calcd Resid. Dens.  1.86A   From C5A             2.82 eA-3
```

Author Response: Although we were unable to determine the presence of several domains we did observe a strong dissagrement in a number of reflections with h<-11. We think that that and the unefficient absorption correction (rather usual in gold compound data) are responsible of the residual densities.

Alert level C

```
PLAT213_ALERT_2_C Atom C31C           has ADP max/min Ratio .....        3.1 prolat
PLAT213_ALERT_2_C Atom C32C           has ADP max/min Ratio .....        3.1 prolat
PLAT213_ALERT_2_C Atom C31B           has ADP max/min Ratio .....        3.1 prolat
PLAT213_ALERT_2_C Atom C32B           has ADP max/min Ratio .....        3.1 prolat
PLAT220_ALERT_2_C NonSolvent   Resd 1  C   Ueq(max)/Ueq(min) Range       4.8 Ratio
PLAT222_ALERT_3_C NonSolvent Resd 1  H   Uiso(max)/Uiso(min) Range       5.1 Ratio
PLAT234_ALERT_4_C Large Hirshfeld Difference C42B     --C41B     .       0.21 Ang.
```

Author Response: This is consequence of the disorder in the PEt3 ligand.

```
PLAT241_ALERT_2_C High   'MainMol' Ueq as Compared to Neighbors of       C22B Check
PLAT241_ALERT_2_C High   'MainMol' Ueq as Compared to Neighbors of       C42B Check
PLAT250_ALERT_2_C Large U3/U1 Ratio for Average U(i,j) Tensor ....        2.3 Note
PLAT342_ALERT_3_C Low Bond Precision on  C-C Bonds ..............    0.01056 Ang.
PLAT790_ALERT_4_C Centre of Gravity not Within Unit Cell: Resd.  #          1 Note
              C18 H23 Au N3 P
PLAT905_ALERT_3_C Negative K value in the Analysis of Variance ...     -0.402 Report
PLAT910_ALERT_3_C Missing # of FCF Reflection(s) Below Theta(Min).          6 Note
PLAT971_ALERT_2_C Check Calcd Resid. Dens.  1.35A   From N3B             2.49 eA-3
```

Author Response: Although we were unable to determine the presence of several domains we did observe a strong dissagrement in a number of reflections with h<-11. We think that that and the unefficient absorption correction (rather usual in gold compound data) are responsible of the residual densities.

```
PLAT971_ALERT_2_C Check Calcd Resid. Dens.  0.81A   From C7B             2.18 eA-3
```

Author Response: Although we were unable to determine the presence of several domains we did observe a strong dissagrement in a number of reflections with h<-11. We think that that and the unefficient absorption correction (rather usual in gold compound data) are responsible of the residual densities.

```
PLAT971_ALERT_2_C Check Calcd Resid. Dens.  1.14A   From Au2             2.02 eA-3
```

Author Response: Although we were unable to determine the presence of several domains we did observe a strong dissagrement in a number of reflections with h<-11. We think that that and the unefficient absorption correction (rather usual in gold compound data) are responsible of the residual densities.

```
PLAT971_ALERT_2_C Check Calcd Resid. Dens.  1.01A   From Au1             1.96 eA-3
```

Author Response: Although we were unable to determine the presence of several domains we did observe a strong dissagrement in a number of reflections with h<-11. We think that that and the unefficient absorption correction (rather usual in gold compound data) are responsible of the residual densities.

```
PLAT971_ALERT_2_C Check Calcd Resid. Dens.  0.26A   From Au2             1.78 eA-3
```

Author Response: Although we were unable to determine the presence of several domains we did observe a strong dissagrement in a number of reflections with h<-11. We think that that and the unefficient absorption correction (rather usual in gold compound data) are responsible of the residual densities.

PLAT971_ALERT_2_C Check Calcd Resid. Dens. 0.79A From C32B 1.72 eA-3

Author Response: Although we were unable to determine the presence of several domains we did observe a strong dissagrement in a number of reflections with h<-11. We think that that and the unefficient absorption correction (rather usual in gold compound data) are responsible of the residual densities.

PLAT971_ALERT_2_C Check Calcd Resid. Dens. 0.78A From P1B 1.65 eA-3

Author Response: Although we were unable to determine the presence of several domains we did observe a strong dissagrement in a number of reflections with h<-11. We think that that and the unefficient absorption correction (rather usual in gold compound data) are responsible of the residual densities.

PLAT971_ALERT_2_C Check Calcd Resid. Dens. 0.74A From Au1 1.58 eA-3

Author Response: Although we were unable to determine the presence of several domains we did observe a strong dissagrement in a number of reflections with h<-11. We think that that and the unefficient absorption correction (rather usual in gold compound data) are responsible of the residual densities.

PLAT971_ALERT_2_C Check Calcd Resid. Dens. 2.06A From C10B 1.54 eA-3

Author Response: Although we were unable to determine the presence of several domains we did observe a strong dissagrement in a number of reflections with h<-11. We think that that and the unefficient absorption correction (rather usual in gold compound data) are responsible of the residual densities.

PLAT972_ALERT_2_C Check Calcd Resid. Dens. 0.81A From P1B -1.93 eA-3

Author Response: Although we were unable to determine the presence of several domains we did observe a strong dissagrement in a number of reflections with h<-11. We think that that and the unefficient absorption correction (rather usual in gold compound data) are responsible of the residual densities.

PLAT972_ALERT_2_C Check Calcd Resid. Dens. 0.74A From Au1 -1.89 eA-3

Author Response: Although we were unable to determine the presence of several domains we did observe a strong dissagrement in a number of reflections with h<-11. We think that that and the unefficient absorption correction (rather usual in gold compound data) are responsible of the residual densities.

PLAT972_ALERT_2_C Check Calcd Resid. Dens. 0.71A From Au1 -1.66 eA-3

Author Response: Although we were unable to determine the presence of several domains we did observe a strong dissagrement in a number of reflections with h<-11. We think that that and the unefficient absorption correction (rather usual in gold compound data) are responsible of the residual densities.

PLAT972_ALERT_2_C Check Calcd Resid. Dens. 0.77A From Au1 -1.57 eA-3

Author Response: Although we were unable to determine the presence of several domains we did observe a strong dissagrement in a number of reflections with h<-11. We think that that and the unefficient absorption correction (rather usual in gold compound data) are responsible of the residual densities.

PLAT977_ALERT_2_C Check Negative Difference Density on H5A -0.31 eA-3
PLAT977_ALERT_2_C Check Negative Difference Density on H10A -0.31 eA-3
PLAT977_ALERT_2_C Check Negative Difference Density on H37B -0.40 eA-3

Alert level G

PLAT002_ALERT_2_G Number of Distance or Angle Restraints on AtSite 11 Note
PLAT083_ALERT_2_G SHELXL Second Parameter in WGHT Unusually Large 60.48 Why ?
PLAT172_ALERT_4_G The CIF-Embedded .res File Contains DFIX Records 12 Report
PLAT230_ALERT_2_G Hirshfeld Test Diff for P1B --C31C . 5.1 s.u.
PLAT230_ALERT_2_G Hirshfeld Test Diff for P1B --C31B . 7.6 s.u.
PLAT301_ALERT_3_G Main Residue Disorder ....(Resd 1) 17% Note
PLAT860_ALERT_3_G Number of Least-Squares Restraints .... 10 Note
PLAT883_ALERT_1_G No Info/Value for _atom_sites_solution_primary . Please Do !
PLAT912_ALERT_4_G Missing # of FCF Reflections Above STh/L= 0.600 64 Note
PLAT933_ALERT_2_G Number of OMIT Records in Embedded .res File ... 11 Note
PLAT965_ALERT_2_G The SHELXL WEIGHT Optimisation has not Converged Please Check
PLAT978_ALERT_2_G Number C-C Bonds with Positive Residual Density. 3 Info

---

1 **ALERT level A** = Most likely a serious problem - resolve or explain
4 **ALERT level B** = A potentially serious problem, consider carefully
30 **ALERT level C** = Check. Ensure it is not caused by an omission or oversight
12 **ALERT level G** = General information/check it is not something unexpected

1 ALERT type 1 CIF construction/syntax error, inconsistent or missing data
34 ALERT type 2 Indicator that the structure model may be wrong or deficient
6 ALERT type 3 Indicator that the structure quality may be low
6 ALERT type 4 Improvement, methodology, query or suggestion
0 ALERT type 5 Informative message, check

It is advisable to attempt to resolve as many as possible of the alerts in all categories. Often the minor alerts point to easily fixed oversights, errors and omissions in your CIF or refinement strategy, so attention to these fine details can be worthwhile. In order to resolve some of the more serious problems it may be necessary to carry out additional measurements or structure refinements. However, the purpose of your study may justify the reported deviations and the more serious of these should normally be commented upon in the discussion or experimental section of a paper or in the "special_details" fields of the CIF. checkCIF was carefully designed to identify outliers and unusual parameters, but every test has its limitations and alerts that are not important in a particular case may appear. Conversely, the absence of alerts does not guarantee there are no aspects of the results needing attention. It is up to the individual to critically assess their own results and, if necessary, seek expert advice.

Publication of your CIF in IUCr journals

A basic structural check has been run on your CIF. These basic checks will be run on all CIFs submitted for publication in IUCr journals (*Acta Crystallographica*, *Journal of Applied Crystallography*, *Journal of Synchrotron Radiation*); however, if you intend to submit to *Acta Crystallographica Section C* or *E* or *IUCrData*, you should make sure that full publication checks are run on the final version of your CIF prior to submission.

Publication of your CIF in other journals

Please refer to the *Notes for Authors* of the relevant journal for any special instructions relating to CIF submission.

PLATON version of 18/09/2020; check.def file version of 20/08/2020

Datablock ev161042_2_0m - ellipsoid plot

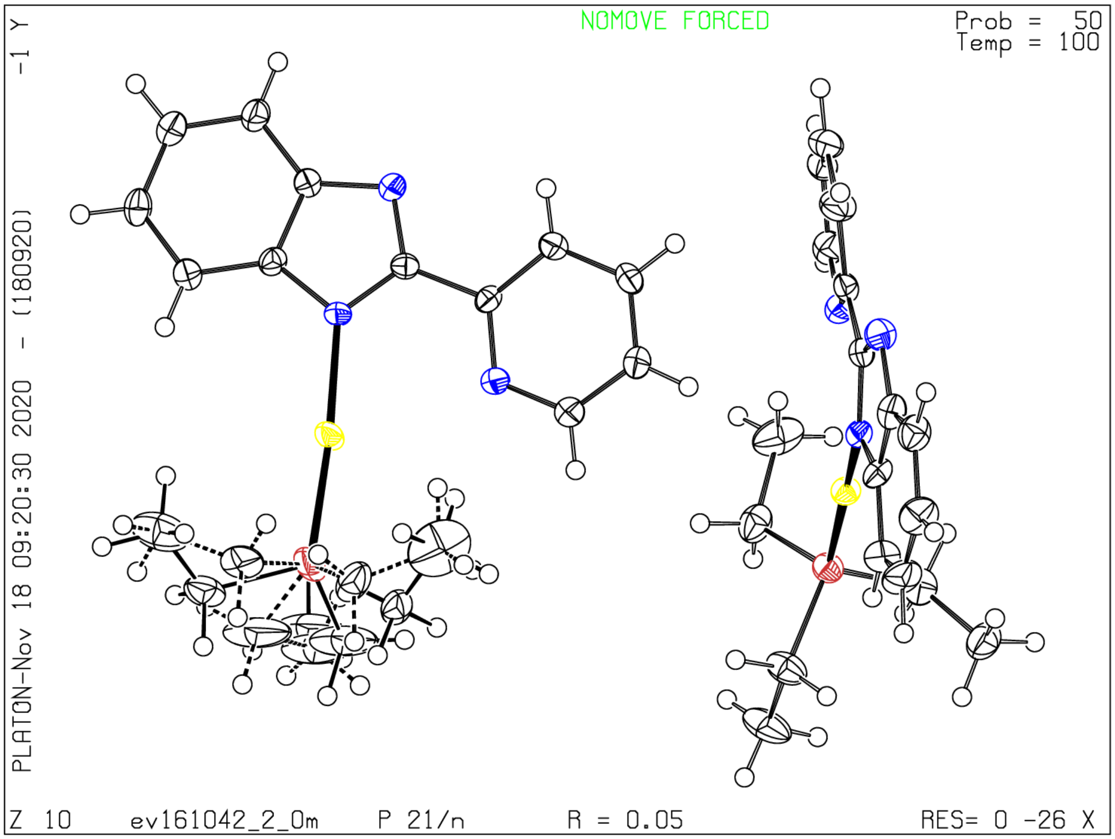

Supplement: Supplementary file 1 [file pharmaceuticals-14-00010-s001.pdf]
